# Emerging zoonoses.

**DOI:** 10.3201/eid0403.980324

**Published:** 1998

**Authors:** F A Murphy

**Affiliations:** School of Veterinary Medicine, University of California, Davis 95616-8734, USA. famurphy@ucdavis.edu

## Abstract

In the past few years, emergent disease episodes have increased; nearly all have involved zoonotic or species-jumping infectious agents. Because there is no way to predict when or where the next important new zoonotic pathogen will emerge or what its ultimate importance might be, investigation at the first sign of emergence of a new zoonotic disease is particularly important. Such investigation may be described in terms of a discovery-to-control continuum: from recognition of a new disease in a new setting to complex phases involving the hard science disciplines pertaining to discovery, the epidemiologic sciences pertaining to risk assessment, and activities pertaining to risk management. Today, many activities involving zoonotic disease control are at risk because of a failed investigative infrastructure or financial base. Because zoonotic diseases are distinct, their prevention and control will require unique strategies, based more on fundamental research than on traditional approaches. Such strategies require that we rebuild a cadre of career-committed professionals with a holistic appreciation of several medical and biologic sciences.


### Emerging Zoonoses

**Frederick A. Murphy**
University of California, Davis, California, USA

In the past few years, emergent disease episodes have increased; nearly all have involved zoonotic or species-jumping infectious agents. Because there is no way to predict when or where the next important new zoonotic pathogen will emerge or what its ultimate importance might be, investigation at the first sign of emergence of a new zoonotic disease is particularly important. Such investigation may be described in terms of a discovery-to-control continuum: from recognition of a new disease in a new setting to complex phases involving the hard science disciplines pertaining to discovery, the epidemiologic sciences pertaining to risk assessment, and activities pertaining to risk management. Today, many activities involving zoonotic disease control are at risk because of a failed investigative infrastructure or financial base. Because zoonotic diseases are distinct, their prevention and control will require unique strategies, based more on fundamental research than on traditional approaches. Such strategies require that we rebuild a cadre of career-committed professionals with a holistic appreciation of several medical and biologic sciences.

In the past few years, emergent disease episodes have increased in the United States and globally. The list of important emergent diseases is impressive indeed and, given what we know about disease ecology, it will only continue to grow. Nearly all of these emergent disease episodes have involved zoonotic infectious agents; that is, they have involved the transmission of the etiologic agent to humans from an ongoing reservoir life cycle in animals or arthropods, without the permanent establishment of a new life cycle in humans. Fewer episodes have involved species-jumping by the etiologic agent; that is, they derive from an ancient reservoir life cycle in animals but have subsequently established a new life cycle in humans that no longer involves an animal reservoir.

### Distinct Prevention and Control Strategies

Nearly all of the major topics for discussion at this conference involve either zoonotic or species-jumping infectious agents. Prevention and control strategies for diseases caused by these agents are different from those required for diseases whose etiologic agent has long relied on human-to-human transmission for its survival. The Centers for Disease Control and Prevention's (CDC) acute infectious disease prevention and control strategies were largely developed from experiences with vaccine-preventable childhood diseases, sexually transmitted diseases, hepatitis, and other diseases for which traditional clinically based or laboratory-based surveillance can provide the base for intervention activities such as vaccination or antimicrobial chemotherapy. For the zoonoses and for diseases caused by species-jumping agents, prevention and control strategies have come from diverse bases. At the heart of this research have been individual scientists who have spent whole careers accumulating highly specialized knowledge and experience. In fact, the work of these scientists might best be described as fundamental research—research seeking the means for disease control and prevention.

### Predicting the Emergence of Zoonotic and Species-Jumping Pathogens

In general, there is no way to predict when or where the next important new zoonotic pathogen will emerge or what its ultimate importance might be. A pathogen might emerge as the cause of a geographically limited curiosity, intermittent disease outbreaks, or a new epidemic. No one could have predicted the emergence or zoonotic

Address for correspondence: Frederick A. Murphy, School of Veterinary Medicine, University of California, Davis, CA 95616-8734, USA; fax: 530-752-2801; e-mail: famurphy@ucdavis.edu.

nature of the bovine spongiform encephalopathy prion in cattle in the United Kingdom in 1986, the emergence or zoonotic potential of Sin Nombre virus as the cause of hantavirus pulmonary syndrome in the Southwest in 1993, and certainly not the species-jumping emergence of HIV as the cause of AIDS in 1981. Consequently, investigation at the first sign of emergence of a new zoonotic disease is particularly important, although the investigation usually resembles a field- and laboratory-based research project rather than a typical case-control–based outbreak investigation. This reality must drive strategic planning for dealing with new zoonotic diseases.

### Factors Contributing to the Emergence of Zoonotic Diseases

Many elements can contribute to the emergence of a new zoonotic disease: microbial/virologic determinants, such as mutation, natural selection, and evolutionary progression; individual host determinants, such as acquired immunity and physiologic factors; host population determinants, such as host behavioral characteristics and societal, transport, commercial, and iatrogenic factors; and environmental determinants, such as ecologic and climatologic influences.

Emergence of new zoonotic pathogens seems to be accelerating for several reasons: global human and livestock animal populations have continued to grow, bringing increasingly larger numbers of people and animals into close contact; transportation has advanced, making it possible to circumnavigate the globe in less than the incubation period of most infectious agents; ecologic and environmental changes brought about by human activity are massive; and bioterroristic activities, supported by rogue governments as well as organized amateurs, are increasing, and in most instances the infectious agents of choice seem to be zoonotic.

### Ecologic Factors Contributing to the Emergence of Zoonotic Diseases, as Exemplified by Arbovirus Diseases

Contributing to the emergence of zoonotic diseases is the capacity of microorganisms and viruses to adapt to extremely diverse and changing econiches. One of the most complex sets of adaptations concerns the arboviruses and their transmission by specific arthropods. When ecosystems are altered, disease problems of humans and animals follow. Population movements and the intrusion of humans and domestic animals into arthropod habitats have resulted in emergent disease episodes, some of which are the stuff of fiction. The classic example is the emergence of yellow fever when humans entered the Central American jungle to build the Panama Canal—many contemporary examples suggest that similar events will continue to occur. Deforestation and settlement of new tropical forest and farm margins have exposed farmers and domestic animals to new arthropods and the viruses they carry. Mayaro and Oropouche virus infections in Brazilian woodcutters who cleared the Amazonian forest in recent years is a case in point. The opening up of isolated ecosystems has contributed to emergent disease episodes. Remote econiches, such as islands, with immunologically naive potential reservoir hosts and vectors are often particularly vulnerable to an introduced virus. For example, the initial Pacific island-hopping of Ross River virus in the 1980s from its original econiche in Australia caused "virgin soil" epidemics of arthritis-myalgia syndrome in Fiji and Samoa—this virus will surely reemerge. Increased long-distance air travel facilitates the movement of infected persons and exotic arthropod vectors around the world. The introduction of the Asian mosquito *Aedes albopictus* to the United States in water contained in used tires represents an unsolved problem of this kind. Increased long-distance livestock transportation facilitates the movement of viruses and arthropods (especially ticks) around the world. The introduction and emergence of African swine fever virus from Africa into the Americas in the 1960s and 1970s seem prophetic; although this virus is not zoonotic (it does not infect humans), this experience should raise the question concerning possible transport of Crimean-Congo hemorrhagic fever virus or other tick-borne pathogens to new locales. Ecologic factors pertaining to uncontrolled urbanization and environmental pollution are contributing to many emergent disease episodes. Arthropod vectors breeding in accumulations of water (e.g., tin cans, old tires) and sewage-laden water are a problem worldwide. Environmental chemical toxicants (herbicides, pesticides, residues) can also affect vector-virus relationships directly or indirectly. Ecologic factors related to expanding primitive irrigation systems are becoming important in virus disease emergence, as exemplified by the emergence of Japanese encephalitis in newly developed rice-growing areas of southern Asia. New routings of long-distance bird migrations, brought about by new man-made water impoundments, represent an important yet still untested risk of introduction of arboviruses into new areas. Global warming, which affects sea level, estuarine wetlands, fresh water swamps, and human habitation patterns, may also be affecting vector-virus relationships throughout the tropics; however, data are scarce and long-term programs to study the effect of global warming have too often not included the participation of tropical medicine experts.

Of all the ecologic factors contributing to arthropod-borne zoonotic viral disease emergence, uncontrolled urbanization is the most important. The mega cities of the tropics, with their lack of sanitary systems, serve as incubators for emerging zoonoses—they represent the most difficult zoonotic disease risks of the next century. Who will pay to control disease in these cities? How will the World Health Organization (WHO) and the Pan American Health Organization (PAHO) serve the needs of the people in these cities? How will CDC serve the interests of the people of the United States in preventing emergent zoonotic diseases from emigrating from these cities? Lessons from the past suggest that we need a larger national and international enterprise to deal with emergent zoonoses in such settings, but even more we need an adaptable enterprise, one that can adjust quickly to diverse episodes.

### Lessons from Venezuelan Equine Encephalitis Epidemics

Past Venezuelan equine encephalitis epidemics provide lessons regarding today's zoonotic disease prevention and control systems. In 1971, as the virus crossed from Mexico into Texas, agricultural disease control authorities were prepared to start shooting and burying horses in a massive slaughter campaign. Scientists from CDC and the Middle America Research Unit (at the time a unit of the National Institutes of Health) provided the virologic and epidemiologic base to override the sanitary rifle strategy of agricultural authorities, and the U.S. Army provided its then new TC83 vaccine. Conflict between agricultural and public health agencies was rampant; if this kind of emergency happened again, the response might not be much different. If the epidemic in Venezuela and Columbia in 1995 had progressed and jumped north, which agency would have stepped forward to direct control activities? What would have been done? Do we have an interagency plan? The same question might be asked in regard to the possible introduction of Rift Valley fever virus into the United States. In my view, our government institutional culture fails in long-term, interdisciplinary, interagency strategy development—we need strategies that are proof-tested to ensure success.

There is another lesson from the 1971 and 1995 Venezuelan equine encephalitis epidemics. Thirty years ago the arbovirus community was large, very experienced in field work and disease control actions, and holistic in perspective and expertise. Arbovirologists were able to bring together all necessary expertise—entomology and vector biology, ecology, mammology, ornithology, epidemiology, and virology. However, today this community, like so many others supporting zoonotic public health programs, is very small, rather poorly experienced in field work, and scientifically fragmented. Experts on mosquito biology, genetics, ecology, and vector competence are becoming more and more separated from the people in local mosquito control agencies who are expected to terminate epidemics. We had better fix this, organizationally and culturally, if we are to deal with mosquito-borne diseases in the 21st century.

### Lessons from the Equine Morbillivirus Outbreak in Australia

Recent experiences in Australia with a new morbillivirus disease add still more lessons in zoonotic disease prevention and control. In 1994, horses on a property in Queensland developed acute respiratory distress with hemorrhagic manifestations—14 of 21 infected horses died. A horse trainer and a stable-hand became ill after nursing a sick horse—the trainer died. The disease was found to be caused by a previously unknown morbillivirus. Remarkably, in 1996 fruit bats (flying foxes) were found to be the natural host of the virus. Studies are under way to unravel these findings.

One lesson is similar to that taught by experiences with Venezuelan equine encephalitis. In Australia, where animal disease research is organized on a national basis but human disease research (and prevention and control activities) on a state basis, this disease was given over to the Australian Animal Health Laboratory. One can imagine the public outcry if it had turned out that humans were at greater risk than horses. Again, cooperation across a wide range of institutions is required to deal with zoonoses, but when human health is at risk, I cannot imagine our public health institutions deferring to animal disease and agricultural institutions. Similar turf issues have been raised in the United States and in the United Kingdom in regard to the recent episode of H5N1 influenza in chickens and humans in Hong Kong.

### Lessons from Ebola Hemorrhagic Fever Epidemics

Should we be concerned about Ebola virus? Is there a risk to Africa that compares with the everyday problems of other zoonoses such as malaria or yellow fever? Is there a risk to people in North America or Europe? If the worst that might happen is an occasional importation resulting in a small cluster of cases, should we be concerned? If the time and place of such episodes are unpredictable, should we not just wait and react after the fact? The risk reflected in these questions is difficult to evaluate because we know so little. However, we can say that as western-style hospitals become more affordable for Africans, nosocomial Ebola amplification will increase, and epidemics will get larger.

These viruses and the diseases they cause need to be understood because the risk they represent is unknown and the risk for future episodes is so unpredictable—the same should be said in regard to all similarly lethal zoonotic pathogens. For example, we need to find the natural reservoir of Ebola virus and learn how its prevalence in its natural environment and how transmission to humans are regulated. In Africa, the emergence of Ebola virus could dramatically increase if its still unknown reservoir host(s) increased, the virus changed its behavior, or ecologic factors brought additional reservoir hosts into play. We need to know enough to detect such changes quickly. The concerned public would not be satisfied if public health leaders decided on a wait-and-see approach for dealing with Ebola hemorrhagic fever or other diseases with similar pathogenic potential.

Dealing with Ebola virus and similar very dangerous infectious agents need not be thought of as so expansive or expensive as to be unrealistic. Field-based epidemiologic studies are needed; diagnostic systems require better placement in laboratories in Africa. Training is a major need—not through short courses, but rather through advanced career training and experience; transcending these is the need for an expanded research base, which in turn requires more national laboratory facilities and resources for work at biosafety level (BSL) 4. These needs must be met in all industrialized countries on behalf of developing countries.

### Lessons from Rabies Epidemics

Rabies provides many lessons in how viral adaptation contributes to emergence in new econiches. Often, the necessary ecologic elements are in place and the recipe for emergence simply involves the introduction of virus; a dramatic illustration was the appearance of epidemic raccoon rabies in the eastern United States. The epidemic was traced to raccoons imported from Florida to West Virginia in 1977—as usual, human perturbation of an ecosystem, in this instance involving the transport of wild raccoons from an endemic site, caused trouble. One key to our understanding of this episode was the discovery that rabies virus is not one virus; rather, it is a set of different genotypes, each transmitted within a separate reservoir host econiche. In North America, there are six terrestrial animal genotypes, including the raccoon virus genotype. Raccoons bite raccoons that bite raccoons, and after some time, their virus becomes a distinct genotype, highly adapted to the host cycle. When the full significance of this discovery was realized, many mysteries of rabies ecology were clarified. The lesson here is that modern virologic research is the key for prevention and control programs such as those carried out by the CDC Rabies Laboratory and the Texas State Health Department, which is achieving much success with its coyote vaccination program.

### Lessons from the Hantavirus Pulmonary Syndrome Epidemic

In 1993, hantavirus pulmonary syndrome was first recognized in the southwestern United States. Cases have been found in 28 states; as of 1997, more than 164 cases had been confirmed in the United States and more than 400 throughout the Americas—the death rate has been approximately 45%. At the beginning of the investigation, serologic tests provided the first clue about the nature of the causative virus. Viral RNA was amplified from patient specimens, and a previously unknown hantavirus, now named Sin Nombre virus, was uncovered. Later, scientists from CDC, the University of New Mexico, and elsewhere found that several variant viruses were distributed over large areas of the United States, all previously unknown, all entrenched in specific rodent reservoirs, all capable of zoonotic transmission to humans.

The laboratory and field work resembled fundamental field- and laboratory-based research, not a traditional outbreak investigation. Sin Nombre virus and its relatives could only be dealt with in laboratories with the most sophisticated molecular biologic and immunologic technologies, the most expert staff scientists, and the kind of global perspective seen in WHO international reference centers. If scientists in these laboratories compete rather than collaborate, how will public health be given priority? How will technology transfer occur as rapidly as needed? How will the full capacity of more specialized biomedical research laboratories be brought to bear?

The tradition of public service holds the answer. When the rabies immunofluorescence test was developed at CDC, it was made available immediately to state and other laboratories. When *Legionella pneumophila* was discovered, cultures and reagents were made available immediately to everyone concerned. This tradition, in turn, has led over the years to the immediate transfer to CDC of new infectious agents isolated in other laboratories—Marburg virus from Germany, Lassa virus from Yale, HIV from France, poliovirus isolates from everywhere. Research competition has never been the point—public health has been the purpose at hand. The perpetuation of this tradition seems extremely important.

### Lessons from the Bovine Spongiform Encephalopathy Epidemic in Cattle and New-Variant Creutzfeldt-Jakob Disease in Humans

Bovine spongiform encephalopathy (BSE) in the United Kingdom may provide more lessons than any other recent emergent zoonotic disease episode. The disease was first diagnosed in the United Kingdom in 1986; as of 1997, more than 170,000 cattle had been reported as infected, but modern statistical methods have indicated that about one million cattle had been infected, roughly half of which entered the human food chain in the United Kingdom.

Today, with the wisdom of hindsight, it might be said that the ministry of agriculture in the United Kingdom failed to react in time to what was clearly a great risk to the livestock and related food industries of the country—every element of its disease prevention and control responsibilities might be called into question. By 1990, the front pages of British newspapers were filled with BSE articles, forcing the question "…does BSE pose a risk to human health?" British government officials responded, "…there is nothing to worry about…" This of course led the public to become more skeptical. The editors of the journal Nature reacted as follows:

> …Never say there is no danger [risk]. Instead, say that there is always a danger [risk], and that the problem is to calculate what it is… Never say that the risk is negligible unless you are sure that your listeners share your own philosophy of life…

In my view, this response sums up one of the central precepts of public health practice.

In 1995, the BSE agent was reported to be the cause of a new human zoonotic disease, new-variant Creutzfeldt-Jakob disease. By 1997, 26 cases had been reported in the United Kingdom and one in France. A recent report from The Royal Society states that there is now a compelling case regarding new-variant Creutzfeldt-Jakob disease as the human manifestation of BSE. With such a small number of cases, it is impossible to predict future numbers of cases of the human disease, but clearly the damage to the livestock and related food industries of the United Kingdom will continue. BSE may be instructive in other ways, especially in its extension into the worlds of macroeconomics, international trade, political science, and even global governance.

In all these lessons, one of the most important points is the need for greater epidemiologic resources and better trained professionals for dealing with human and animal diseases or with the zoonotic interface between the two. This training component requires consideration of all steps along the discovery-to-control continuum.

### The Discovery-to-Control Continuum as Applied to Zoonotic Diseases

Initial investigation at the first sign of emergence of a new zoonotic disease must focus on practical characteristics such as death rate, severity of disease, transmissibility, and remote spread, all of which are important predictors of epidemic potential and societal risk. Various elements of a discovery-to-control continuum are usually called for: discovery, the recognition of a new zoonotic disease in a new setting; epidemiologic field investigation; etiologic investigation; diagnostics development; focused research; technology transfer; training and outreach; and ultimately control, elimination, and eradication. Of course, not all of these elements are appropriate in every emerging zoonotic disease episode—decisions must be made and priorities must be set.

In the initial phases in the discovery-to-control continuum, people outside the “citadel” (the traditional federal community of investigators and officials) must be recognized—local clinicians, pathologists (including medical examiners and forensic pathologists), veterinarians and animal scientists, ecologists, wildlife scientists, as well as local public health officials, many of whom have not been enamored of their experiences in dealing with those inside the citadel. The important early role of primary diagnostic laboratories and the reference laboratory networks that support them must also be recognized. In this era of the primacy of molecular microbiology and virology, it bears reminding that many of the early investigative activities surrounding the identification of a possibly emergent zoonotic disease must be carried out in the field, not in the laboratory. This is the world of shoe-leather epidemiology (the logo of CDC’s Epidemic Intelligence Service program is the outline of the sole of a shoe with a prominent hole worn in it), as well as of molecular microbiology and virology.

In the intermediate phases in the discovery-to-control continuum, the continuum progresses to the general area of risk management, the area represented not by the question what’s going on here? but by the question what are we going to do about it? This phase may include expansion of many elements: technology transfer involving diagnostics development and proof testing, vaccine and drug development and proof testing, sanitation and vector control, and medical and veterinary care activities and their adaptation to the circumstances of the disease locale; commercialization, where appropriate, of diagnostics, vaccines, and therapeutic agents in quantities needed and provision of these materials through nongovernment organizations or government sources; training, outreach, continuing education, and public education, each requiring professional expertise and adaptation to the special circumstances of the disease locale; and communications, employing the technologies of the day such as the Internet and professional expertise.

Further along the discovery-to-control continuum, activities become more complex. Frustration often occurs at intermediate points as administrators and politicians drag their feet in regard to resource allocation. This frustration, in turn, drives scientists back to their laboratories, to the world of research, to the front end of the continuum. Younger scientists, particularly, become cynical of the harsh political world of risk management, even though this is the arena in which their discoveries must prove themselves.

More expensive and specialized expertise and resources come into play in the final phases of the discovery-to-control continuum: public health systems, including rapid case-reporting systems, surveillance systems, vital records and disease registers, staffing and staff support, logistic support, legislation and regulation, and expanded administration; special clinical systems, including isolation of cases, quarantine, and patient care; and public infrastructure systems, including sanitation and sewerage, safe food and water supplies, and reservoir host and vector control.

The question of facilities needs in the United States is an element of our capacity to fulfill the discovery-to-control continuum. What about BSL-3+ and BSL-4 laboratory facilities west of the Appalachians? Recent debate makes it clear that having two BSL-4 facilities in the United States (CDC in Atlanta, and the U.S. Army Medical Research Institute of Infectious Diseases in Frederick, Maryland) and one in Canada (at the new center in Winnipeg) is not enough. Plans for a few small BSL-4 labs in U.S. academic centers may help in expanding basic research supported by competitive grants, but they will not support expanded field-based research. Which government agency will step forward to solve this problem? And in a related way, which government agency will step forward to solve the unique problem of career-committed professional personnel needs for dealing with emerging zoonotic diseases?

### Conclusions

Who will be the world's doctor? Who will be the world's expert on zoonotic diseases? These questions are taken from an editorial in the New York Times, May 12, 1995. It seems that many authorities, including those at CDC, are saying that they have the answer to these questions in regard to all emerging diseases. Their answers have been in the form of proposals and funding requests to expand global disease surveillance, diagnostics, communications, and emergency response systems, a global training program, and a global stable funding base. However, somewhat distinct strategies are needed to deal specifically with emerging zoonotic diseases, and these strategies have not been fully developed. Examples have been given in this paper to suggest that these strategies must involve more of a field and laboratory research enterprise than a traditional surveillance and reference diagnostics enterprise. In some cases, it is not even clear who might do the focused applied research that must underpin advances in zoonotic disease prevention and control. In present circumstances, where the survival of institutions is at stake, turf battles are exacerbated, and competition rather than cooperation between academic institutions and government agencies ensues. CDC may be getting new funds, but there is no parallel sense of "good times ahead" out in the country. This is happening in contradiction to public expectations. Data clearly show that the concerned public wants more disease control and intervention actions, more of the medical research needed to drive such actions, and more participation across the country. Numerous surveys of public opinion done by Research!America show that the concerned public is willing to pay. In my view, public expectations can only be met by the integration of the nascent global public health emerging infectious disease network, with networks focused on threats posed by livestock animal diseases, crop plant diseases, and bioterrorism. The public would see such an overall system as having a high benefit:cost ratio, which would solve several high priority problems most efficiently.

Frederick Murphy is professor of virology at the School of Veterinary Medicine, University of California, Davis. He has served as director of the Division of Viral and Rickettsial Diseases as well as director of the National Center for Infectious Diseases, Centers for Disease Control, and dean of the School of Veterinary Medicine, UC Davis. His professional interests include the pathogenesis and ultrastructural pathology of viral diseases, viral characterization and taxonomy, rabies, arboviruses, viral hemorrhagic fevers, viral encephalitides, public health policy, vaccine development, and new and reemerging infectious diseases.

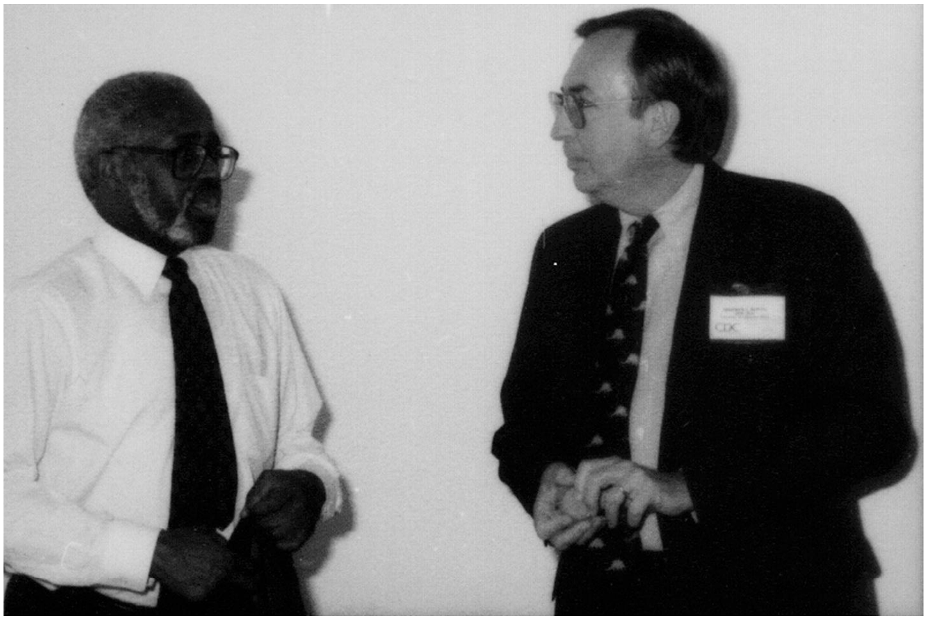

George Hill (L)
Meharry Medical College,
Nashville, Tennessee, USA
Frederick A. Murphy
University of California,
Davis, California, USA

